# Adenovirus-mediated delivery of Sema3A alleviates rheumatoid arthritis in a serum-transfer induced mouse model

**DOI:** 10.18632/oncotarget.19915

**Published:** 2017-08-03

**Authors:** Yue Teng, Zhanhai Yin, Jing Li, Kun Li, Xu Li, Yan Zhang

**Affiliations:** ^1^ Center for Translational Medicine, The First Affiliated Hospital of Xi'an Jiaotong University, Xi’an, Shaanxi 710061, People’s Republic of China; ^2^ Department of Orthopaedics, The First Affiliated Hospital of Xi’an Jiaotong University, Xi’an, Shaanxi 710061, People’s Republic of China; ^3^ Clinical Laboratory, Shanxi Mineral Hospital, Xi’an, Shaanxi 710014, People's Republic of China

**Keywords:** Sema3A, rheumatoid arthritis, serum-transfer induced mouse model, macrophages

## Abstract

Rheumatoid arthritis is a chronic autoimmune disease characterized by infiltration of inflammatory cells into the synovium and destruction of cartilage and bone. Macrophages, fibroblast-like synoviocytes (FLS), and osteoclasts are critical cells driving the pathogenesis of RA. Semaphorin 3A (Sema3A) is recently identified as an essential player in the bone homeostasis, however its role in RA progression especially in the macrophage polarization are poorly understood. In the present study, we found that Sems3A levels were significantly decreased in RA serum and synovial fluid compared to OA controls. There was a negative correlation between Sema3A levels and RA severity. Using *in vitro* cell cultures, we showed for the first time that Sema3A promoted IL-4 induced M2 macrophage polarization, whereas prohibited LPS/IFN-γ induced M1 polarization. Sema3A inhibited VEGF-induced endothelial cells proliferation and migration, suppressed VEGF-mediated invasion and IL-6 production of FLS while stimulating their apoptosis. In addition, Sema3A retarded osteoclastogenesis. *In vivo* data demonstrated that Sema3A administration attenuated joint tissue damage and the severity of experimental arthritis. Our findings uncovered Sema3A as a promising diagnostic biomarker and novel prevention and treatment strategies in arthritis treatment.

## INTRODUCTION

Rheumatoid arthritis (RA) is a debilitating chronic autoimmune disease characterized by dysfunctional cellular and humoral immunity, enhanced migration and attachment of peripheral macrophages and inflammatory leukocytes to the synovium and articular cartilage of diarthrodial joints [1]. RA is the most common chronic inflammatory arthritis, affecting 0.5-1% of the adult population worldwide with 5-50 per 100,000 people newly developing the condition annually. Women are affected 3-5 times as frequently as men [2]. Besides the dysfunctional joints, RA also leads to a severely debilitating form with pulmonary, renal and cardiovascular involvement. In 2010 it resulted in about 49,000 deaths globally, and in 2013 it resulted in 38,000 deaths [3–5].

The joints of RA patient are characterized by an infiltration of inflammatory cells, including macrophages into the synovium, leading to chronic inflammation, pannus formation and subsequent irreversible cartilage and bone destruction [6]. Macrophages are one of the most abundant cell types in RA synovium and cartilage/pannus junction. Being a major source of various pro-inflammatory cytokines and chemokines, macrophages play pivotal roles in the progression of RA [7]. The abundance and activation of macrophages is markedly elevated in the synovial tissues of RA patients and positively correlated with disease severity [8]. Elimination of macrophages also alleviates the symptoms of collagen-induced arthritis in mice [9].

Synovial angiogenesis could not be neglected in maintaining and promoting RA [10]. The levels of vascular endothelial growth factor (VEGF), which is the main signaling protein involved in angiogenesis, was significantly elevated in RA synovial fluids and tissues, as well as serum. Its level correlates closely with the disease activity of RA, particularly with the numbers of swollen joints [11]. VEGF targets several kinds of cells in the RA joints, including endothelial cells (ECs) lining the blood vessels and fibroblast-like synoviocytes (FLS) [12].

Osteoclasts, which are widely present in subchondral bone and the pannus-bone interface in RA samples, are the cells responsible for cartilage and bone resorption in the late stage of RA [13]. Osteoclast progenitors arise from both circulating cells and local cells developed within the synovial tissues and subchondral bone. Inflamed synovial tissues provide cellular sources of receptor activator of NF-κB ligand (RANKL), the strong inducer of osteoclastogenesis. RANKL synergizes with pro-inflammatory cytokines in the inflamed synovium, including TNF-α, IL-1β, IL-6 and IL-17, immune complexes, and toll-like receptor activators, to promote osteoclastogenesis and bone resorption [14].

Semaphorin 3A (Sema3A), a membrane-associated secreted protein and diffusible axonal chemorepellent, has been recently identified as an essential player in the bone homeostasis maintenance [15, 16]. Sema3a knockout mice exhibit severe osteopenic phenotypes with dramatic decrease in bone mass [15]. Sema3A exerts its osteoprotective effect by suppressing osteoclast differentiation and increasing osteoblastic bone formation synchronously [15]. Although Takagawa and colleagues have revealed that the mRNA and protein expression of Sema3A in synovial lining cells was decreased in RA tissues compared with OA controls [17], the role of Sema3A in RA still remains obscure.

Our previous studies demonstrated that the receptor for Sema3A, neuropillin-1 (Nrp1), plays essential roles in stabilizing discoidin domain receptor 2 (DDR2) and promoting osteoclastogenesis [18, 19]. These facts prompted us to investigate that whether Sema3A could be exploited as a diagnostic maker and therapeutic avenue for RA. In this study, we detected the serum and synovial fluid levels of Sema3A in RA samples. Then we examined the effects of Sema3A on macrophages, ECs, FLS, and osteoclasts, which are indispensable cells involved in RA progression. Finally, we used the serum-transfer induced arthritis (STA) model to investigate the therapeutic potential of Sema3A in RA. Our findings demonstrated for the first time that Sema3A functions as an immunosuppressive factor in RA and is a promising diagnostic and therapeutic target in the prevention and treatment of RA.

## RESULTS

### Sema3A levels negatively correlated with RA activity

**Figure 1:**
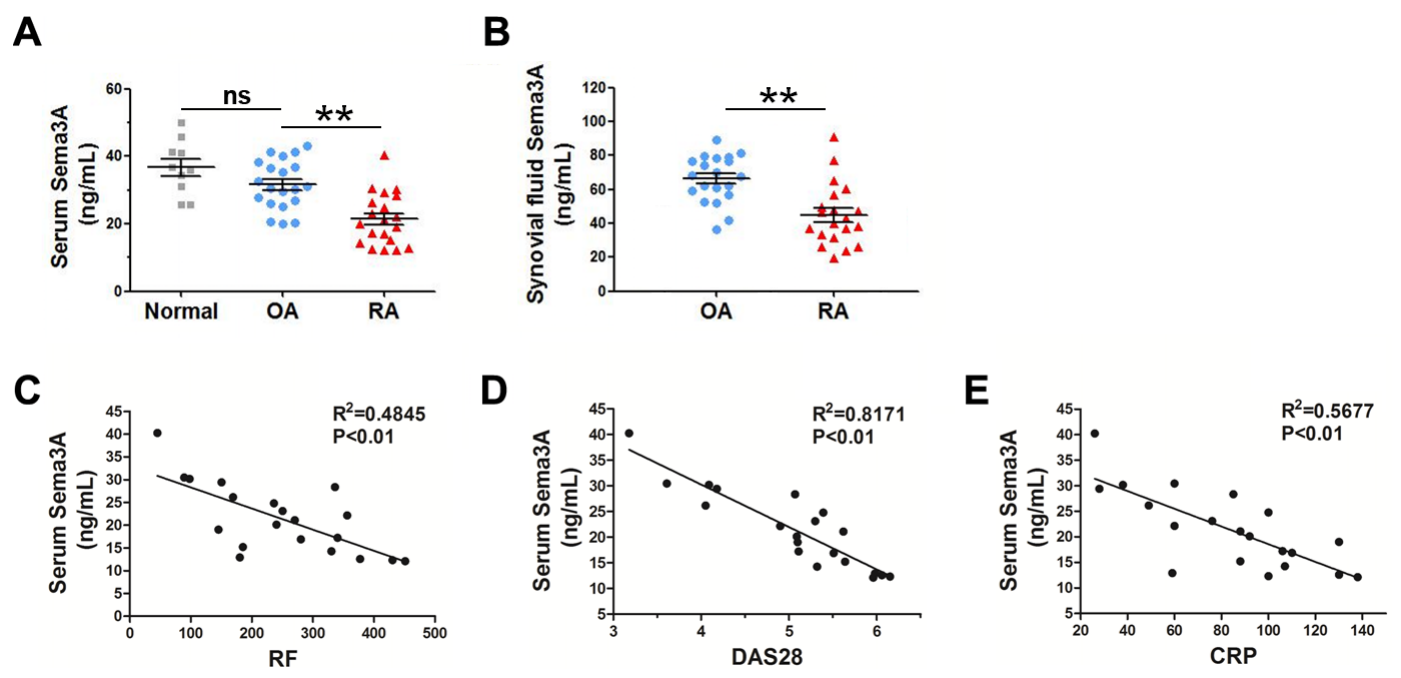
The serum and synovial fluid levels of Sema3A are significantly decreased in RA samples **(A)** Serum levels of Sema3A in normal, OA, and RA samples. **(B)** Synovial fluid levels of Sema3A in RA samples. **(C)** The correlation of serum Sema3A levels with RF. **(D)** The correlation of serum Sema3A levels with DAS28. **(E)** The correlation of serum Sema3A levels with CRP. ** P< 0.01.

It was reported that Sema3A expression was decreased in RA synovial tissues compared with OA controls [17]. However there is no literature until now demonstrating the Sema3A level in serum or synovial fluid of RA patients. Here, we collected 20 RA and 20 OA samples, and compared the levels of Sema3A in the serum and synovial fluid of RA and OA patients. As shown in Figure 1A, the serum levels of Sema3A were significantly decreased in RA samples compared with OA controls. The synovial fluid levels of Sema3A were consistent with that of serum (Figure 1B). When analyzing the serum Sema3A levels with RA activity indicators such as rheumatoid factor (RF), disease activity score 28 (DAS28), C-reactive protein (CRP), we found a significant negative correlation between serum Sema3A levels and these RA activity indicators (Figure 1C-1E). The detailed clinical profile of clinical samples was displayed in Table 1. These findings demonstrated that secreted Sema3A levels are significantly downregulated in RA patients, and are negatively related to RA progression.

**Table 1:** Profile of patients enrolled in this study

	RA patients (n=20)	OA patients (n=20)
**Demographics**		
Age (years, mean±SD)	61.35±9.68	69.35±6.55
Gender (No. Male/No. Female)	7/13	7/13
**Clinical characteristics**		
Disease duration (years, mean±SD)	10±3.71	7.79±2.51
RF (IU/mL, mean±SD)	247.85±115.01	NA
CRP (mg/L, mean±SD)	83.5±33.34	15.7±7.06
DAS28 (mean±SD)	5.07±0.84	NA
**Medications**		
Aminoglucose, n (%)	16 (80%)	20 (100%)
Sodium hyaluronate, n (%)	8 (40%)	11 (55%)
NSAID, n (%)	16 (80%)	13 (65%)
TCM, n (%)	9 (45%)	8 (40%)
DMARDs, n (%)	9 (45%)	NA
Glucocorticoid, n (%)	13 (65%)	NA
Anti-TNF-α therapy, n (%)	4 (20%)	NA
Tocilizumab, n (%)	3 (15%)	NA

RF: rheumatoid factor; CRP: C-reactive protein; DAS28: disease activity score in 28 joints; NSAID: nonsteroidal anti-inflammatory drug; TCM: traditional Chinese medicine; DMARDs: disease-modifying antirheumatic drugs; TNF-α: tumor necrosis factor-α; NA: not applicable.

### Sema3A promoted macrophage repolarization from M1 to M2 phenotype

**Figure 2:**
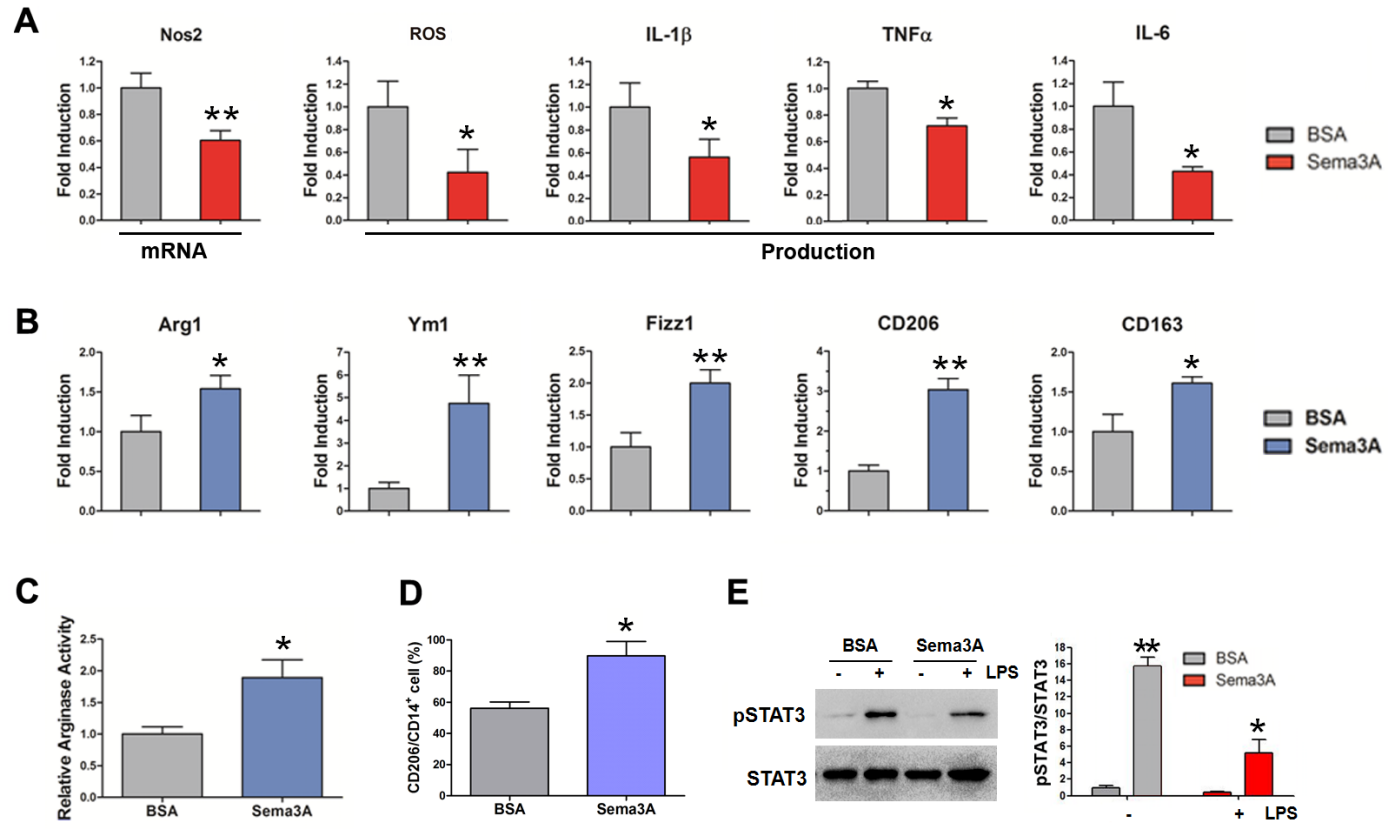
Sema3A promotes macrophage repolarization from M1 to M2 phenotype **(A)** The mRNA expression of iNos2, and the production of ROS, IL-1β, TNF-α, and IL-6 were significantly reduced after Sema3A treatment. **(B)** The mRNA expression of Arg1, Fizz 1, Ym1, CD206, and CD163 after Sema3A treatment. **(C)** The activity of arginase was increased after Sema3A stimulation. **(D)** The percentage of CD206 positive cells. **(E)** The LPS-induced activation of STAT3 in the presence of Sema3A. * P< 0.05, ** P< 0.01.

Based on our observation, we supposed that Sema3A functions as a protective factor in RA progression. To verify this hypothesis we detected the effect of Sema3A on macrophages polarization, given the essential and distinct roles of M1/M2 macrophages in RA initiation, development and resolution. We pretreated bone marrow macrophages (BMMs) with recombinant Sema3A or BSA (as controls) for 24 h and then further stimulated BMMs with M1 inducers lipopolysaccharide (LPS) and interferon-γ (IFN-γ) or M2 inducer IL-4 for 4 h, followed by mRNA and production analysis of M1 or M2 markers respectively. As shown in Figure 2A, the M1 macrophage markers including inducible nitric oxide synthase 2 (iNos2) mRNA expression, reactive oxygen species (ROS) production, and secretion of cytokine IL-1β, IL-6, TNFα were significantly declined due to Sema3A treatment. On the contrary, pretreatment of Sema3A before M2 induction boosted the elevation of the M2 mRNA expression, including Arginase 1 (Arg1), found in inflammatory zone 1 (Fizz 1), Ym1, CD206, and CD163 (Figure 2B). The activity of arginase was increased after Sema3A treatment (Figure 2C). The CD206 expression on the cell surface was elevated (Figure 2D, Supplementary Figure 1). The results from RAW264.7 cell were consistently with that of BMMs (Supplementary Figure 2). To investigate the underlying mechanism, we detected the activation of signal transducer and activator of transcription 3 (STAT3), an important signaling in M1 polarization, in the presence of Sema3A. Western blotting results indicated that the phosphorylation of STAT3 was decreased after Sema3A stimulation (Figure 2E). These findings indicated that Sema3A could promote M1 macrophage transit to M2 phenotype at least *in vitro*.

### Sema3A restrained the function of endothelial cells and fibroblast-like synoviocytes

**Figure 3:**
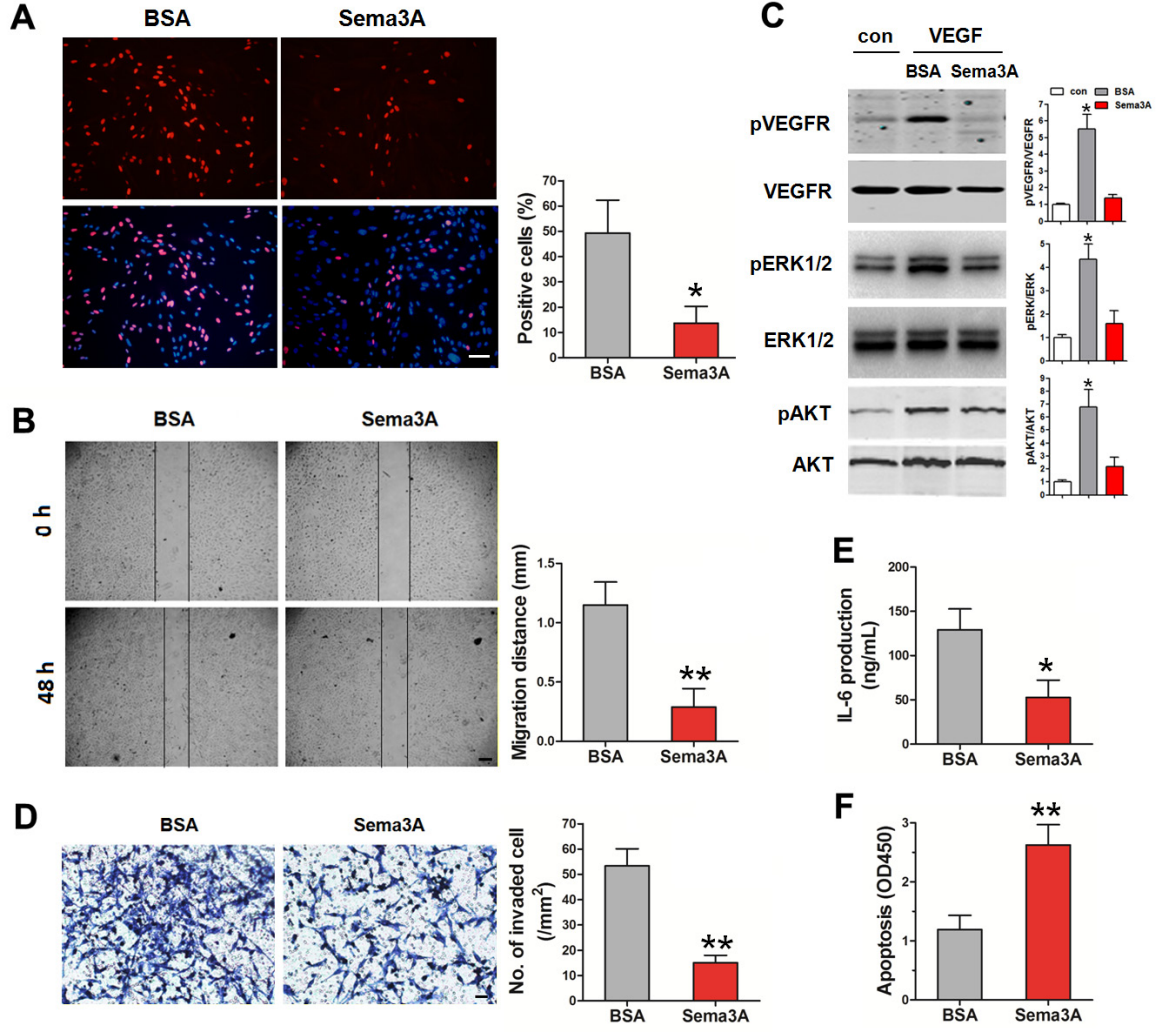
The effects of Sema3A on ECs and FLS **(A)** The proliferation of HUVECs in the presence of Sema3A was significantly decreased, Bar, 250 μm. **(B)** The inhibition of Sema3A on the migration of HUVECs Bar, 300 μm. **(C)** The phosphorylation of ERK1/2 and AKT of HUVECs in the absence or presence of Sema3A. **(D)** Invasion of FLS induced by VEGF_165_, Bar, 20 μm. **(E)** The production of IL-6 in FLS after Sema3A treatment. **(F)** Sema3A enhances the serum starvation induced apoptosis of FLS. * P< 0.05, ** P< 0.01.

Besides macrophages, ECs and FLS also play important roles in RA development. Therefore, we examined the effect of Sema3A on ECs and FLS. Proliferation and migration of HUVECs induced by VEGF_165_ were detected in the absence or presence of Sema3A. As indicated in Figure 3A, Sema3A treatment dramatically suppressed the proliferation of HUVECs induced by VEGF_165_. Wound healing assay also demonstrated the inhibition of Sema3A on the VEGF_165_-induced migration of HUVECs (Figure 3B). The phosphorylation of ERK1/2 and AKT induced by VEGF_165_ was attenuated in the Sema3A-treated HUVECs (Figure 3C). Invasion abilities of FLS induced by VEGF_165_ were assessed by matrigel invasion assays. As expected, Sema3A treatment significantly decreased the invasion of FLS (Figure 3D). The production of IL-6 after VEGF_165_ induction, an indicator of FLS immune function, was also reduced due to Sema3A treatment (Figure 3E). Moreover, Sema3A could enhance the serum starvation induced apoptosis of FLS (Figure 3F). Taken together, these results indicated that Sema3A inhibited ECs proliferation migration, and activation, suppressed FLS function while stimulated their apoptosis.

### Sema3A inhibited osteoclastogenesis

**Figure 4:**
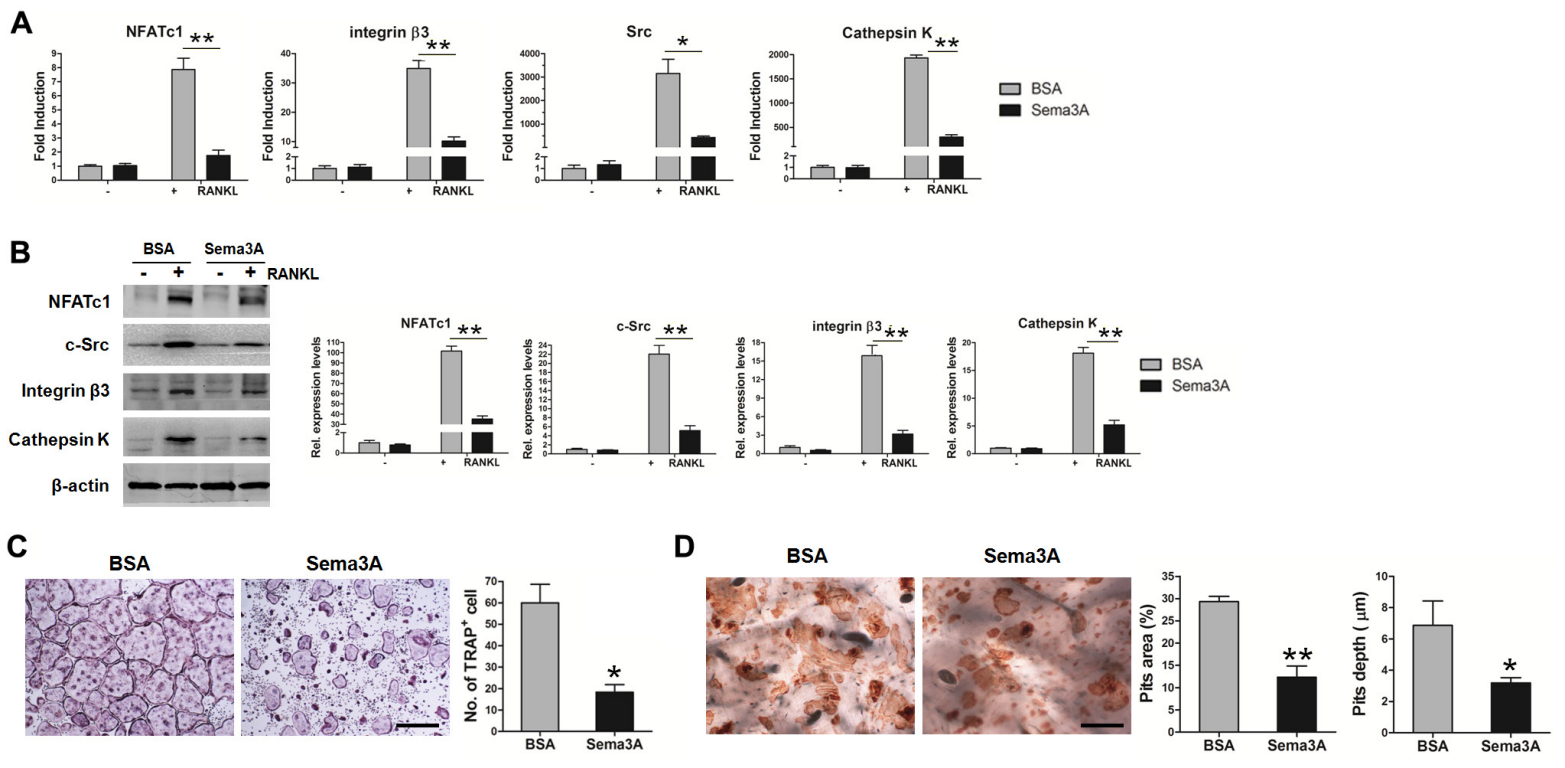
Sema3A inhibits osteoclast differentiation and function **(A)** The mRNA levels of the osteoclast differentiation markers. **(B)** The protein levels of the osteoclast differentiation markers. **(C)** TRAP staining of osteoclasts, Bar, 500 μm. **(D)** Pits assay in the absence or presence of Sema3A, Bar, 250 μm. * P< 0.05, ** P< 0.01.

Previous studies indicated that Sema3A inhibits osteoclast differentiation via Nrp1, but the effect of Sema3A on osteoclast function still remains unclear [15]. Therefore, we detected the RANKL-induced differentiation and bone resorption of BMM when Sema3A was added. As expected, in the presence of Sema3A, the osteoclast differentiation was retarded, as demonstrated in the reduced mRNA levels (Figure 4A) and protein expression (Figure 4B) of marker genes, including NFATc1, integrin β3, c-Src, and Cathepsin K. The number of TRAP positive osteoclasts was also diminished (Figure 4C). When we induced mature osteoclasts on bone slices and stained the pits with lectin, we observed that the pits area and depth were also significantly decreased in the presence of Sema3A (Figure 4D). These findings indicated that Sema3A not only inhibits osteoclast differentiation but also suppresses osteoclast function.

### Sema3A delayed RA development in a K/BxN serum-transfer induced arthritis mouse model

**Figure 5:**
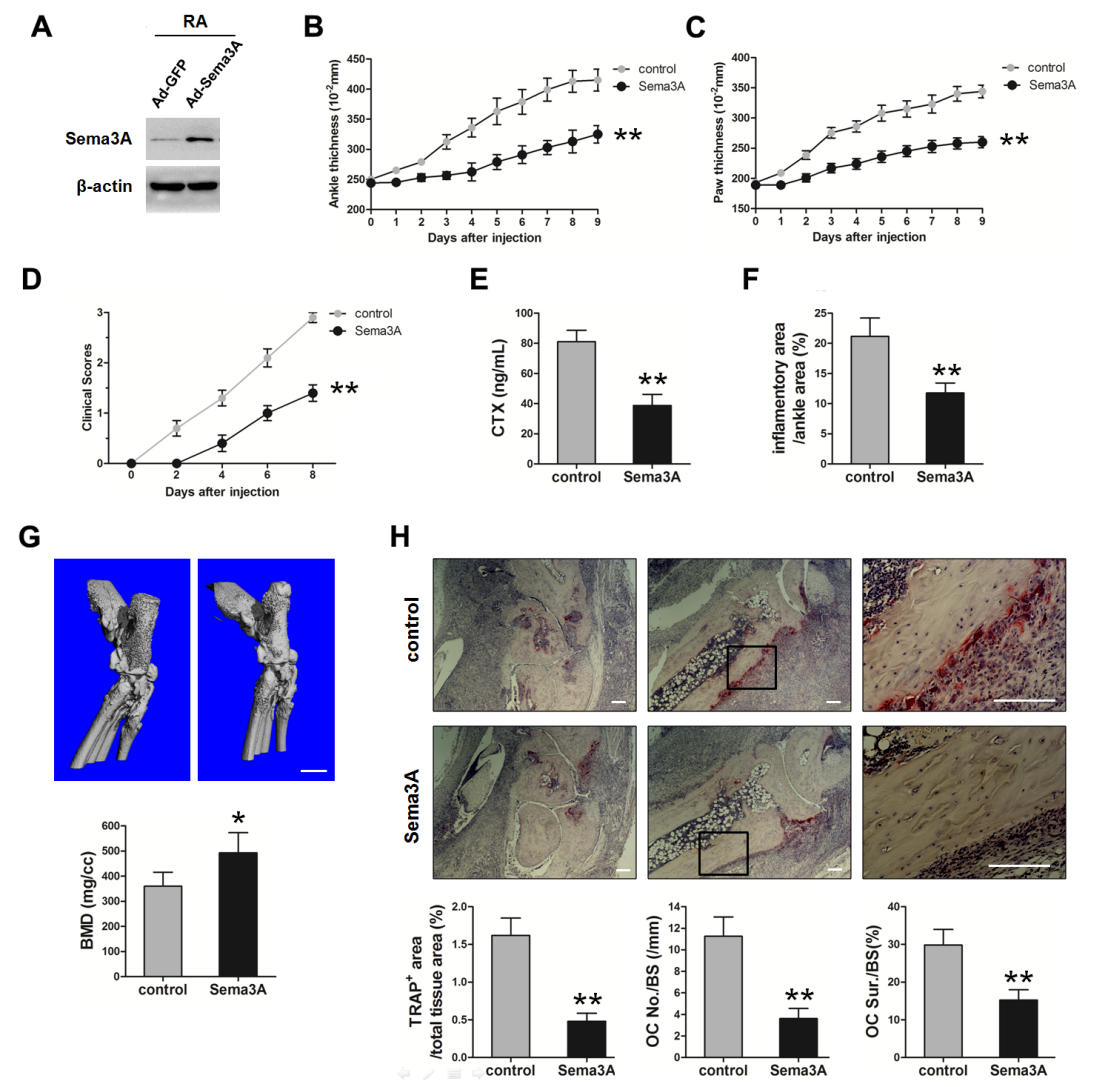
*In vivo* validation of Sema3A function in the STA mouse model **(A)** The validation of Sema3A expression level in joint tissues. **(B)** The thickness of ankle from control and Sema3A-treated mice. **(C)** The thickness of hind paw. **(D)** The clinical scores of the hind limbs. **(E)** The serum levels of CTX in control and Sema3A-treated mice. **(F)** The inflammatory area was decreased in the Sema3A-treated mice. **(G)** Micro-CT analysis of ankle joints of control and Sema3A-treated mice, Bar, 1 mm. **(H)** TRAP staining of ankle joints sections from control and Sema3A-treated mice. The right panel is enlarged from the middle panel, Bar, 50 μm. * P< 0.05, ** P< 0.01.

Given the observation that Sema3A regulated the function of macrophages, ECs, FLS, and osteoclasts, which are important cells involved in the pathogenesis of RA, we examined the effect of Sema3A administration in RA progression using the STA mouse model. The expression of Sema3A protein in joint tissues was verified by Western Blotting (Figure 5A). Obvious swelling was observed on day 2 post injection in control mice. As shown in Figure 5B and C, the thickness of ankle and hind paw were gradually increased, reaching the peak at day 9, with around 4.2 and 3.5 mm respectively in the control mice. However, the onset of RA in Sema3A-treated mice was delayed, the thickness of ankle and hind paw were significantly attenuated. There was remarkable differences between Sema3A-treated and control mice in the clinical scores of the hind limbs (Figure 5D), in consistent with the measurement of ankle and paw thickness. The serum CTX of Sema3A-treated mice was also significant decreased, suggesting the possible change of bone resorption (Figure 5E). Also, the inflammatory area was decreased in the Sema3A-treated mice (Figure 5F). Micro-CT analysis further revealed that the bone resorption was obviously diminished and the bone mineral density (BMD) was significantly improved in the ankle joints of Sema3A-treated mice (Figure 5G). Moreover, TRAP staining of ankle joints sections from control mice exhibited obvious osteoclasts and bone erosion, that of Sema3A-treated mice were remarkably reduced (Figure 5H). These observations indicated that Sema3A delayed RA development and severity in the STA mouse model.

## DISCUSSION

RA is a chronic disease that affects approximately 1% of the world population, characterized by a massive infiltration of a variety of inflammatory cells into the synovium, leading to the destruction of cartilage and bone [20]. These cells include macrophages, T-cells, B-cells, neutrophils, dendritic cells, fibroblasts, osteoclasts, and chondrocytes [21].

Macrophages account for the majority of inflammatory cells in the RA synovial [22]. They possess broad pro-inflammatory, destructive, and remodeling capabilities, not only actively participating in but also critically driving the progression of RA [8, 22]. Macrophages secrete inflammation promoting cytokines (e.g., IL-6, IL-1β, and TNF-α), growth factors (e.g., granulocyte macrophage colony stimulating factor, GM-CSF), and chemokines. These pro-inflammatory macrophages in the diseased condition have been depicted as M1 macrophages. Alternatively, macrophages secrete IL-4 and Arg1, which are anti-inflammatory and promote tissue repair, are termed as M2-phenotype [22–24]. Activation of certain signaling pathways in synovial macrophages, including nuclear factor kappa-light-chain-enhancer of activated B cells (NF-κB), STAT3, and phosphoinositide 3-kinase (PI3K) signaling pathways, enhances the M1 phenotype and the inflammatory conditions of the RA synovium [24]. Accumulating evidence suggest that there is an imbalance between M1 and M2 macrophages in RA joints [8, 9]. Strategies targeting synovial macrophages have been proposed for RA treatment, such as reducing their number or viability [8, 9]. However, these are not as ideal as expected because of the important reparative role of macrophages in the resolution process of inflammation [8]. Therefore, regulating their activation status, especially editing macrophage polarization, from inflammatory M1 to anti-inflammatory M2 macrophages, is emerging as a promising therapeutic solution for RA [9]. Using *in vitro* culture of macrophages with Sema3A recombinant protein, our original results demonstrated that Sema3A inhibited LPS/IFN-γ induced M1 polarization of macrophages, whereas promoted IL-4 induced M2 polarization. This finding provided a possibility that Sema3A could be exploited as a macrophage editor for the therapeutic purpose of RA. We further indicated that the moderation of STAT3 signaling may be one of the mechanisms of Sema3A in macrophage repolarization.

Nrp1, the receptor of Sema3A, also recognizes VEGF_165_ as its ligand [25, 26]. VEGF is the most potent proangiogenic factor in RA synovial angiogenesis [27]. VEGF_165_ induces complex formation between Nrp1 and VEGFR2 to enhance VEGFR2 signaling in endothelial cells, leading to increase in endothelial cell proliferation and migration [25]. Due to the co-receptor characteristic of Sema3A and VEGF_165,_ it is reasonable that Sema3A may regulate angiogenesis via competition with VEGF_165_ for binding to Nrp1. There is evidence that Sema3A modulates pathological angiogenesis in mice. For example, Sema3A inhibits vascular regeneration in a mouse model of oxygen-induced retinopathy (OIR) [28], and prevents tumor angiogenesis by stimulating endothelial cell apoptosis and normalizing the pericyte coverage of tumor vessels [29]. However there is no report demonstrating the role of Sema3A in RA angiogenesis. Our results revealed for the first time that Sema3A suppressed endothelial cells proliferation and migration, and inhibited their intracellular signaling, including ERK and AKT activation. In addition to ECs, VEGF also stimulates FLS survival, invasion and their inflammatory function, and protects the apoptotic death of FLS, leading to synovial hyperplasia and progressive bone destruction [11]. VEGF induces FLS produce cytokines such as TNF-α and IL-6. On the other hand, IL-6 can act synergistically with TNF-α and IL-1 to induce VEGF production in FLS, forming a positive feedback [10, 12]. Our results further demonstrated that Sema3A also inhibited VEGF-induced FLS invasion, production of IL-6, as well as stimulating serum starvation induced apoptosis of FLS. Theses roles of Sema3A in endothelial cells and FLS may account to their VEGF antagonistic effect.

The STA model exhibits many clinical and histopathologic similarities of RA, it is a valuable tool in studying the involvement of macrophages in RA due to its distinct immunological characteristics [30]. The arthritic transgenic K/BxN mice are generated by crossing T-cell receptor (TCR) transgenic KRN mice on a C57BL/6 background with autoimmune-prone non-obese diabetic (NOD) mice. Transfer of serum from K/BxN mice into a recipient mouse leads to a rapid and robust onset of inflammatory arthritis, with an incidence of 100% in the same background (C57BL/6) mice. The inflammatory response in the STA model is driven by autoantibodies against the ubiquitously expressed self-antigen, glucose-6-phosphate isomerase (G6PI), leading to the activation of innate immune cells including macrophages, neutrophils, and possibly mast cells [30]. We used this STA model to test the role of Sema3A in RA, our *in vivo* data demonstrated that Sema3A administration protected the progression of arthritis, and significantly decreased the osteoclastogenesis and protected the joint tissue damage. We revealed the potential of Sema3A as a promising avenue for the future treatment of RA.

Our results indicated that Sems3A levels were significantly dropped in RA samples compared to OA controls. There is a negative correlation between Sema3A levels and RA severity. There are multiple sources for Sema3A in the inflammatory joints of RA patients. For example, Sema3A is expressed in all skeletal lineages cells, including osteoclasts, osteoblasts, and osteocytes, and some types of immune cells, such as B-cells, T-cells, macrophages, and dendritic cells [31]. Sema3A is expressed and secreted into the local environment and afterward the bloodstream, making it a promising biomarker for monitoring the RA activity.

It was reported that Nrp1 exerts immunosuppressive effects by activating T-reg cells and enhancing responses to TGF-β1 (a powerful immunoinhibitory cytokine) [32]. Also, Nrp1 could be used as a marker of T-reg cells [33]. Therefore, it is possible that Sema3A function as an immunosuppressive regulator by forming a complex with Nrp1 and plexin-A4 to activate an immunoinhibitory response. This hypothesis is consistent with our results about the role of Sema3A in editing macrophage polarization and antagonizing VEGF-induced effects. Although our *in vitro* data provides some evidence, it still needs to be proved *in vivo*. We will isolate the macrophages in the inflammatory joints to analysis their polarized population using FACS. Moreover, we will detect the specific markers using immunofluorescence or other methods.

To sum up, in the present study we found that Sems3A levels were significantly dropped in RA samples compared to OA controls. There is a negative correlation between Sema3A levels and RA severity. To further identify the involvement of Sema3A in RA progression, we tested the effects of Sema3A on macrophages, ECs, FLS, and osteoclasts, which are important cells participating in RA pathogenesis. Using *in vitro* cell cultures, we showed for the first time that Sema3A promoted IL-4 induced M2 macrophage polarization, whereas prohibited LPS/IFN-γ induced M1 polarization. Further evidence indicated that Sema3A inhibited endothelial cells proliferation and migration, suppressed FLS function while stimulated FLS apoptosis, retarded osteoclastogenesis, playing a protective role in RA in a pleiotropic manner. Our *in vivo* data demonstrated that Sema3A administration attenuated joint tissue damage and the severity of experimental arthritis. Thus, these findings can lead to the development of Sema3A as a novel prevention and treatment strategies in arthritis.

## MATERIALS AND METHODS

### Clinical samples

This study was approved by The Ethics Committee of The First Affiliated Hospital of Xi'an Jiaotong University. Forty diagnosed arthritis patients and ten healthy people in The First Hospital of Xi’an Jiaotong University were enrolled in this study. Informed consent was obtained from all participants. Peripheral blood and synovial fluid were collected and centrifuged at 5000 rpm for 10 min at 4°C. The supernatants were transferred to fresh tubes and stored at -80°C before analysis.

### Sema3A quantification and treatment

The serum and synovial fluid Sema3A concentration was determined using a commercial ELISA kit (LifeSpan Biosciences, WA, USA) according to the manufacturer’s instructions. Recombinant Sema3A protein was obtained from ThermoFisher. The concentration of Sema3A in the cell experiments was 100 ng/mL unless otherwise indicated.

To induce M1 polarization, we treated macrophages with 1 ng/mL LPS and 10 U/ mL IFN-γ for 4 h, while 1 ng/mL IL-4 for 4 h for M2 polarization induction.

### Cell culture

Primary human umbilical vein endothelial cells (HUVECs) were purchased from American Type Culture Collection (ATCC). HUVECs were maintained in Vascular Cell Basal Medium and Endothelial Cell Growth Kit-BBE (ATCC).

Primary bone marrow macrophages (BMMs) were prepared as described previously [34]. Briefly, marrow extracted from femora and tibiae of 6- to 8-wk-old mice were cultured in alpha-Minimum Essential Medium Eagle (α-MEM) containing 10% fetal bovine serum (FBS), 100 IU/mL penicillin, and 100 μg/mL streptomycin, 100 ng/mL macrophage colony-stimulating factor (M-CSF). After 3 days of culture, cells were induced with 100 ng/mL RANKL and 30 ng/mL M-CSF to differentiate into osteoclasts.

Murine RAW264.7 (ATCC) was cultured in Dulbecco’s modified Eagle's medium (DMEM) containing 10% FBS and 1% penicillin/streptomycin.

Primary FLS were isolated as described previously [35]. Synovial tissues were obtained from joint replacement surgery. The tissues were rinse with cold phosphate buffered saline (PBS) and minced to less than 1 mm^3^, followed by incubation in RPMI containing collagenase VIII (500 μg/mL) for 90 min at 37°C (gently mixing every 15 min). After filtering with a 70 μm strainer, the cells were centrifuged, washed, and resuspend at 1×10^6^ cells/mL in 15 mL of DMEM containing 10% FBS, 100 IU/mL penicillin, and 100 μg/mL streptomycin in a T75 flask. The next day, the nonadherent cells were removed and fresh medium was changed every other day until 90% confluence. Passage 3-9 were used for the following experiments.

### Quantitative real-time polymerase chain reaction (qPCR)

Total RNA was extracted using TRIzol (Invitrogen) according to the manufacturer’s instructions. cDNA was synthesized from 2 μg of total RNA using the SuperScript II First-Strand Synthesis System (Invitrogen). The levels of mRNA transcripts were determined by qPCR using the cDNA as the template, specific primers, and the standard SYBR Green PCR Master Mix (Takara) in a CFX96 Sequence Detection System (Bio-Rad). The comparative threshold cycle method was used for relative quantification, and GAPDH gene was used as an endogenous control. Primer sequences are listed in Supplementary Table 1.

### Migration and invasion

Cells were cultured until they reached subconfluence, the monolayer was scratched with a 200 μL pipette tip. Cells were washed with PBS twice and cultured in FBS-free medium for additional 48 h. Cell migration into the wound area was monitored and analyzed using Leica LAS EZ software. Cell invasion experiments were performed using Bio-Coat cell migration chambers (BD Biosciences) with 8-μm-diameter. Cells resuspended in FBS-free medium were added to the Matrigel-coated upper chamber, and 20% FBS medium was placed in the lower chamber. After 48 h the invading cells on the upper surface were wiped with a cotton-tipped applicator, the invading cells on the under surface were fixed with 5% glutaraldehyde fixative and stained with Giemsa.

### Western blotting

Cells were lysed in radioimmunoprecipitation assay (RIPA) buffer (50 mM tris-HCl pH 7.4, 150 mM NaCl, 0.25% deoxycholic acid, 1% NP-40, 1 mM EDTA, 1 mM phenylmethylsulfonyl fluoride, 1 mg/ml aprotinin, and 1 mg/ml leupeptin). Protein concentrations were determined using the bicinchoninic acid (BCA) assay kit (Pierce). Protein was resolved by 10-12% sodium SDS-PAGE and transferred to Hybond-ECL nitrocellulose membranes (Amersham Biosciences). Membranes were blocked with 5% skim milk in tris-buffered saline at room temperature for 1 hour, and then probed with indicated antibodies (1:1000) at 4°C overnight and incubated with the appropriate horseradish peroxidase (HRP)-conjugated secondary antibody. Immunoblots of protein bands were visualized with an enhanced chemiluminescence (ECL) detection kit (Amersham). The used antibodies were as follows: Rabbit anti-Sema3A, Rabbit anti- pSTAT3 (Y705), Mouse anti-STAT3, Mouse anti-β-actin (Abcam), Mouse anti-NFATc1 (Santa cruz), Rabbit anti-c-Src, Rabbit anti-integrin β3 (Cell Signaling), Rabbit anti-cathepsin K (Invitrogen).

### TRAP staining

Cells were fixed with 4% formaldehyde at room temperature for 30 min. After 3 times of PBS wash, cells were stained using an Acid Phosphatase, Leukocyte (TRAP) Kit (Sigma) according to the manufacturer’s instruction.

### Pits assay

Cells were seeded on bone slices and cultured in the inducing medium for 6 days. Then bone slices were incubated in 0.5 N NaOH for 1 min and cells were scraped off using a teeth brush. After fully washing with PBS, the bone slices were incubated with 20 mg/mL peroxidase-conjugated wheat germ agglutinin in PBS (Sigma) for 1 h, and exposed to SIGMAFAST^TM^ 3,3'-Diaminobenzidine tablets (Sigma) for 10 min.

### Micro-CT

The paws of the mouse were fixed in 10% neutral buffered formalin and scanned by a Scanco μCT40 scanner (Scanco Medical AG, Bassersdorf, Switzerland). A threshold of 250 was used for evaluation of all scans. The projection images were reconstructed into a three-dimensional structure with a voxel size of 18 mm and analyzed by VG studio Max2.2 software.

### RA model

The STA mouse model was used to confirm the *in vitro* results. All procedures were performed in accordance with the Xi’an Jiaotong University Animal Care and Use Committee. 8-week-old C57BL/6 mice were housed in a facility with stable humidity and temperature and a 12-hour light-dark cycle. After the mice were anesthetized, arthritis was induced by intraperitoneal (i.p.) injection of 200 μL of serum from arthritic K/BxN or control mice on days 0 and 2. ViraPower™ Adenoviral Expression System (Invitrogen) was used for recombinant adenovirus package. Briefly, Sema3a or Gfp cDNA was inserted into the pAd/CMV/V5-DEST Gateway^®^ Vector, which was under the control of the CMV promoter. Then the digested recombinant vector was transfected into the 293A producer cell line to prepare a crude viral lysate. The adenovirus was amplified, purified, and tittered according to the manufacturer’s instruction. Adenovirus containing Sema3a or Gfp was injected (10^9^ plaque forming unit) into the proximal end of the tibia on day 0 and 5. Hind paw and ankle thickness were measured bilaterally every day (n = 5 per group). Clinical scores were determined on alternate days upon evidence of redness and swelling (0, no arthritis; 1, mild arthritis, foot maintains V-shape; 2, moderate arthritis, foot no longer maintains V-shape; 3, severe arthritis). On day 9, mice were euthanized and blood was collected for the serological examination. Hind limbs were fixed and scanned by μCT.

### Serum CTX quantification

Mice were starvation for 6 h. Then serum CTX-1 concentration was determined using a commercial ELISA kit (LifeSpan Biosciences, WA, USA) according to the manufacturer’s instructions to assess the extent of bone resorption.

### Histology and histomorphometry

The hind paw and ankle were fixed with 10% neutral buffered formalin, followed by decalcification in 14% EDTA for 10 days, paraffin embedding, and H&E and TRAP staining. The histomorphometric parameters of histologic sections including OC number, and OC surface per bone surface were analyzed using BioQuant OsteoII (BioQuant Image Analysis Corporation, Nashville, TN) in a blinded fashion.

### Statistical analysis

All data are representative of at least three independent experiments. Data are expressed as mean ± SEM unless noted otherwise. Correlations between clinical parameters and Sema3A were determined using Spearman’s correlation. All the other experiments were analyzed using 2-tailed unpaired Student’s t test for 2 groups by Prism (GraphPad Software). P < 0.05 was considered significant.

## SUPPLEMENTARY MATERIALS FIGURES AND TABLE


