# Supplementary material for: Adenovirus-mediated delivery of Sema3A alleviates rheumatoid arthritis in a serum-transfer induced mouse model
Source: Oncotarget. 2017 Aug 3;8(39):66270–80. doi: 10.18632/oncotarget.19915 (PMC5630410; doi:10.18632/oncotarget.19915)
Supplement: Supplementary file 1 [file oncotarget-08-66270-s001.pdf]

Adenovirus-mediated delivery of Sema3A alleviates rheumatoid arthritis in a serum-transfer induced mouse model

SUPPLEMENTARY MATERIALS

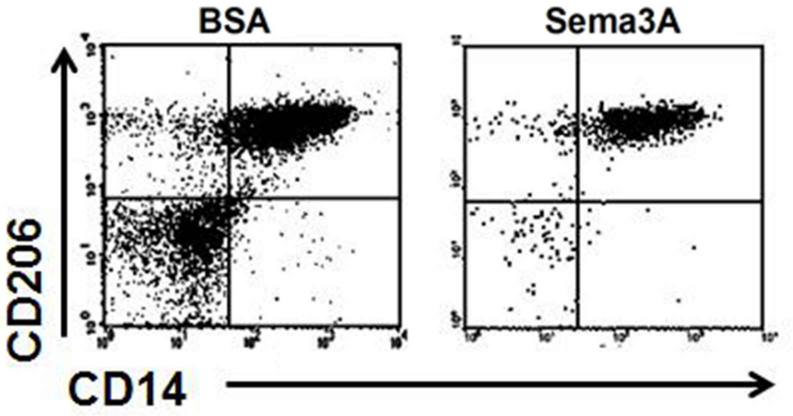

Supplementary Figure 1: The percentage of CD206 positive cells determined by FACS.

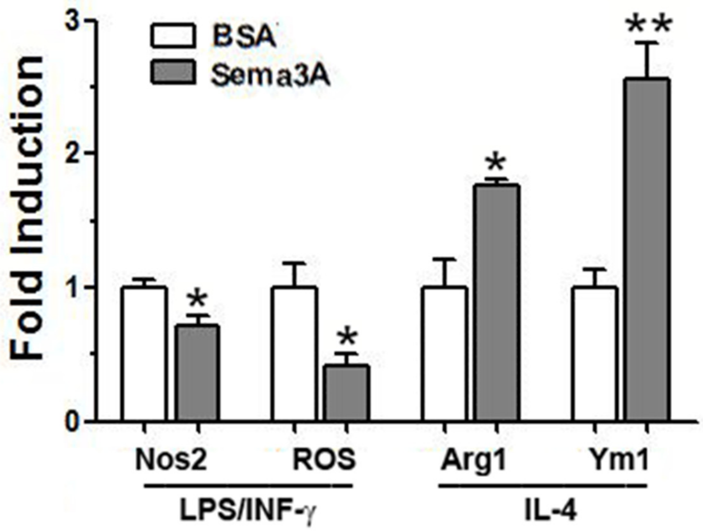

Supplementary Figure 2: The M1 markers were significantly reduced, while M2 markers were increased after Sema3A treatment in RAW264.7 cells. * P< 0.05, ** P< 0.01.

Supplementary Table 1: Primer sequences for PCR analysis

Gene		Primer sequence
Nos2	forward	5'-TCCCAGCACAAAGGGCTCAA-3'
	reverse	5'-TGCGGACCATCTCCTGCATT-3'
Arg1	forward	5'-CAGAAGAATGGAAGAGTCAG-3'
	reverse	5'-CAGATATGCAGGGAGTCACC-3'
Ym1	forward	5′-GGGCATACCTTTATCCTGAG-3′
	reverse	5′-CCACTGAAGTCATCCATGTC-3′
Fizz1	forward	5'-GGTCCCAGTGCATATGGATGAGACCATAGA-3'
	reverse	5'-CACCTCTTCACTCGAGGGACAGTTGGCAGC-3'
CD206	forward	5'-CCTGGAGAGCCAAGCCATGA-3'
	reverse	5'-CCTTTCCGGCAGCCTTTGTC-3'
Nfatc1	forward	5'-CCCGTCACATTCTGGTCCAT-3'
	reverse	5'-CAAGTAACCGTGTAGCTGCACAA-3'
Integrin β3	forward	5'-GATGACATCGAGCAGCTGAAAGAG-3'
	reverse	5'-CCGGTCATGAATGGTGATGAGTAG-3'
Src	forward	5'-CCATCCAGGCTGAGGAGTGG-3'
	reverse	5'-AGGAAGGTCCCTCTCGGGTT-3'
CathepsinK	forward	5'-ATGTGGGTGTTCAAGTTTCTGC-3'
	reverse	5'-CCACAAGATTCTGGGGACTC-3'
Gapdh	forward	5'-AGGTCGGTGTGAACGGATTTG-3'
	reverse	5'-TGTAGACCATGTAGTTGAGGTCA-3'
